# Modification Effect of Nano-Clay on Mechanical Behavior of Composite Geomaterials: Cement, Nano-Silica and Coastal Soft Soil

**DOI:** 10.3390/ma15248735

**Published:** 2022-12-07

**Authors:** Yaying Wang, Wei Wang, Yinuo Zhao, Na Li, Jiale Luo, Asefa Mulugeta Belete, Jiang Ping

**Affiliations:** School of Civil Engineering, Shaoxing University, Shaoxing 312000, China

**Keywords:** nano-material, cement soil, compressive strength, shear strength, ductility index, Duncan–Chang model

## Abstract

To study the modification effect of nano-clay and nano-SiO_2_ on cement-reinforced coastal soft soil, the effects of the nano-SiO_2_ and nano-clay on the mechanical properties of cement soil were studied through unconfined compressive and unconsolidated undrained shear tests, and the Duncan–Chang model was used to fit the test results. Results show that adding nano-clay and nano-SiO_2_ to cement soil improved its compressive and shear strength. The compressive strength and shear strength increased by 18–57% and 3–32%, respectively, with the increase in nano-clay content in a content range of 0–10%. Additionally, nano-clay can enhance the ductility of cement soil. Moreover, nano-clay and nano-SiO_2_ improve the shear strength by increasing the internal friction angle by 1°–2° and cohesion of 9–25%, and the cement-stabilized coastal soft soil enhanced by nano-SiO_2_ and nano-clay conforms to the Duncan–Chang model well.

## 1. Introduction

A large area of marine soft soil is distributed in coastal areas. Due to the characteristics of coastal soft soil, such as large thickness, high compressibility, low shear strength, low permeability and large variation of soil properties [1,2,3,4], buildings built on soft soil may cause uneven settlement and other problems due to insufficient bearing capacity of soft soil. In order to avoid such engineering quality problems and ensure the safety of buildings, coastal soft soil should be reinforced [5].

Currently, coastal soft soil is reinforced by adding 6–12% cement to form coastal cement soil in order to enhance mechanical properties [6,7,8]. However, the use of cement may bring some environmental problems. The dissolution of alkaline substances in cement will bring about the salinization of soil and groundwater, resulting in the reduction of land use area and the pollution of water resources, which will bring inconvenience and harm to people’s lives [9,10,11,12]. In addition, cement also has mechanical defects such as easy cracking, low tensile strength, dry shrinkage and engineering deformation during hydration [13,14]. Therefore, in order to further improve the defects of coastal cement soil, it is of great significance to develop cement-based materials with higher strength and environmental characteristics for infrastructure construction in coastal areas.

At present, many scholars at home and abroad have carried out much research on the use of nano-materials to modify coastal soft soil and proved its effectiveness [4,15,16,17,18]. Compared with traditional materials, nano-materials have the characteristics of great specific surface area and minimal size, which have the ability to speed up cement hydration during the hydration reaction to effectively enhance the mechanical strength and durability of cement soil [19,20], and reduce environmental pollution, providing great possibilities for the sustainable development of the construction industry. Nano-SiO_2_ is a nontoxic and pollution-free material with a high pozzolanic activity, which can improve the strength and durability of cement soil. At the same time, Xiong [21] and Li et al. [22] found that a certain amount of SiO_2_ positively improved the mechanical properties of cement soil. However, an excessive amount of nano-SiO_2_ would lead to the material’s mechanical strength decline. The strength of cement soil can be further improved by adding nano-clay to reinforce coastal soft soil. Experimental studies by Mirgozar [23] and Hamed [24] found that nano-clay could enhance the compactness of cement soil structure and improve the crack resistance and compressive resistance of cement soil, which was an excellent admixture. Among the constitutive models of soil, the Duncan–Chang model can clearly reflect the nonlinear state of soil and show the trend of soil stress–strain, so it is widely used. Zhao et al. [25] found that the Duncan–Chang model can well reflect the stress–strain relationship of fiber-reinforced soil by studying the mechanical properties of fiber-reinforced soil. Gao et al. [26] studied the optimal dosage of rubber powder-modified clay through the Duncan–Chang model. Therefore, the Duncan–Chang model can be fitted with the measured data to accurately describe the nonlinear relationship between the stress and strain of cement soil + nano-SiO_2_ + nano-clay, providing a reference for practical projects.

To sum up, there are few studies on the properties of nano-material composite-modified cement soil, so it is of practical significance to study the properties of nano-composite-modified cement soil. The main purpose of this paper is to investigate the reinforcement effect of nano-clay on cement-stabilized nano-SiO_2_-modified coastal soft soil through unconfined compressive and unconsolidated undrained shear tests, and determine the optimal content of nano-clay and use the Duncan–Chang model to describe the nonlinear state of the mixture, to provide a reference for practical engineering.

## 2. Materials and Experimental Method

### 2.1. Materials

The used coastal soft soil throughout the test was collected from a construction site with a depth of 5–20 m in the city of Shaoxing. Table 1 displays the fundamental mechanical and physical characteristics of the soil. The cement is Lanting P.O32.5 cement. Table 2 shows its basic physical performance indexes. The nano-SiO_2_ with 99.9% purity was purchased from Nanjing Haitai Co., Ltd. Its appearance is white particles, the average particle size is 20 nm and the specific surface area is 200 m^2^/g. The nano-clay is produced by China Hubei Jinxi Montmorillonite Technology Co., Ltd., (Zhongxiang, China) the appearance is light pink powder, the montmorillonite content is more than 96%, its apparent density is 0.45 g/cm^3^ and diameter–thickness ratio is 200. The used water is tap water.

### 2.2. Experiment Scheme

In this paper, nano-SiO_2_ and nano-clay were used to modify cement soil. As shown in Table 3, the experiment scheme is as follows. In the test, the cement content was set as 10% of the dry soil mass, the nano-SiO_2_ content was 4.5‰ of the dry soil mass and the nano-clay content was 0%, 4%, 6%, 8% and 10% of the dry soil mass, respectively. The samples were named SCSC0, SCSC4, SCSC6, SCSC8 and SCSC10 according to the nano-clay content. The water content was 50% of the mixture mass. The samples were cured in water for 7 days and then taken out for testing. The samples were kept in water to simulate soil conditions belowthe underground waterline.

### 2.3. Sample Preparation

**Figure 1:**
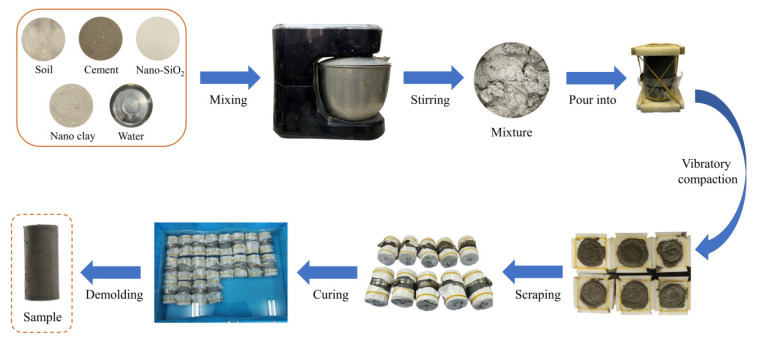
Sample preparation process.

The test was carried out according to the Geotechnical Test Standard (GB/T 50123-2019). Samples for unconfined compressive and unconsolidated undrained shear tests measured 39.1 mm in diameter and 80 mm in height. According to the experiment plan, the pre-set soil, cement, nano-SiO_2_, nano-clay and water were mixed and stirred for 10 min to ensure uniform mixing of the materials. A cylindrical hollow mold with a height of 80 mm and a diameter of 39.1 mm was taken, a plastic film was bound to the bottom of the mold and the mold was tightened with a hoop. Epoxy resin plates were fixed at both ends of the mold. The bottom epoxy resin plate was a solid plate, and the upper epoxy resin plate was a hole-retaining plate with a diameter of 39.1 mm. The mixture was introduced into the mold three times from the upper plate, and the mold was vibrated 40 times after each pour of the mixture to ensure no bubbles in the sample. After three compactions, the samples were placed in the curing box for 6 h. When the sample had a certain strength, the epoxy resin plates on both ends of the mold were removed, and the upper and lower surfaces were scraped flat. The upper and lower surfaces of the sample were wrapped with filter paper, and then the samples with the mold were put into water for curing for 7 days. After reaching the curing time, the sample was taken out from the mold for testing (Figure 1).

### 2.4. Experiment Methods

The unconfined compressive test was carried out using the 01-LH0501 automatic tester produced by China TKA Technology Co., Ltd., (Nanjing, China) The loading rate was set to 1 mm/min, and the test was stopped by 15% axial strain. The unconsolidated undrained shear test was conducted using the TKA-TTS-3S automatic triaxial apparatus produced by Nanjing TKA Technology Co., Ltd. The confining pressures were set to 100 kPa, 200 kPa, 300 kPa and 400 kPa, respectively, and the loading rate was set to 1 mm/min. The test was stopped by 15% axial strain.

## 3. Results and Discussion

### 3.1. Deformation Characteristics

**Figure 2:**
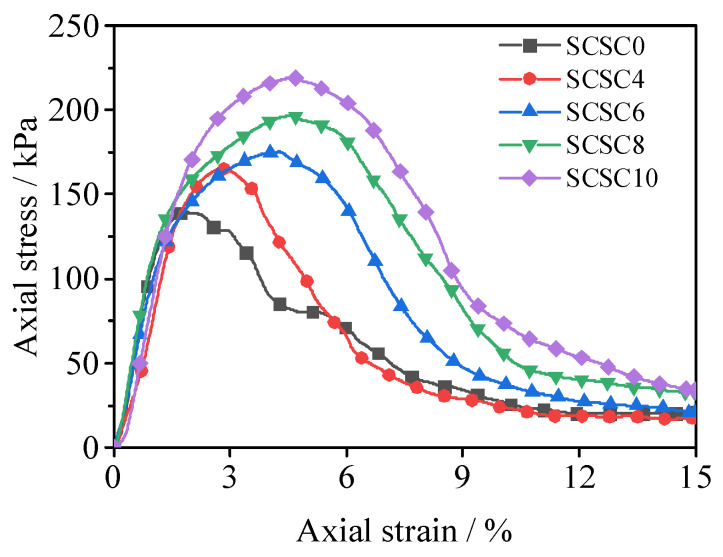
Unconfined compressive strength stress–strain curves of SCSC.

Figure 2 shows the stress–strain curve of the compressive strength of SCSC samples. According to the figure, all stress–strain curves for cement soil with different nano-clay contents soften with increasing nano-clay content. Based on the variation of the stress–strain curve, the process of unconfined compression can be broken down into four stages. The first stage is the initial compression stage, in which the sample is just in contact with the press head, and the stress is minor. The second stage is the elastic deformation stage, in which the stress increases rapidly with the increase in strain, and the curve increases approximately linearly. The third stage is the plastic deformation stage, in which the stress increases slowly with the increase in strain and then reaches the peak. At this time, cracks begin to appear in the sample. However, when the amount of nano-clay grows, the strain value corresponding to the maximum axial strain rises, indicating that the addition of nano-clay can significantly increase soil ductility. The softening stage is the fourth stage. After reaching the peak strength, the downward trend of the curve slows down with the increase in nano-clay content and maintains a certain residual strength. The reason is that nano-clay is a nano-derivative of montmorillonite, which has hydrophilic properties and weak expansibility. Nano-clay adsorbs water to expand and fill the internal pores of the sample. The nucleation effect of nano-clay promotes the cement hydration reaction inside the sample [27], which strengthens the toughness of cement soil, improves its brittleness and effectively improves crack resistance.

#### 3.1.1. Unconfined Compressive Strength and Residual Strength

**Figure 3:**
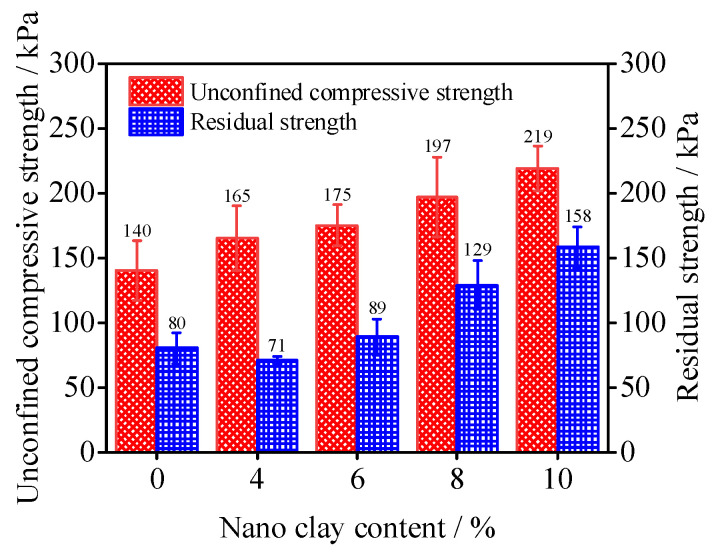
Unconfined compressive strength and residual strength diagram of SCSC sample.

Figure 3 shows the unconfined compressive strength and residual strength of SCSC samples. As can be seen from the figure, the unconfined compressive strengths of SCSC4, SCSC6, SCSC8 and SCSC10 are 165 kPa, 175 kPa, 195 kPa and 219 kPa, which increase by 18%, 25%, 41% and 56% compared with that of SCSC0, respectively. The peak strength of the sample gradually increases as the amount of nano-clay material rises. It can be concluded that in the 0–10% nano-clay content range, the incorporation of nano-clay can improve the compressive strength of the sample, and the strength of the sample with 10% nano-clay reaches the maximum.

When nano-clay content increases, residual strength changes. The residual strengths of SCSC6, SCSC8 and SCSC10 are 89 kPa, 129 kPa and 158 kPa, respectively, which is 11%, 61% and 98% higher than that of SCSC0. However, the residual strength of SCSC4 is 13% lower than that of SCSC0, which indicates that the residual strength of the samples was only improved by the incorporation of nano-clay at 6% or more.

#### 3.1.2. Ductility Index

**Figure 4:**
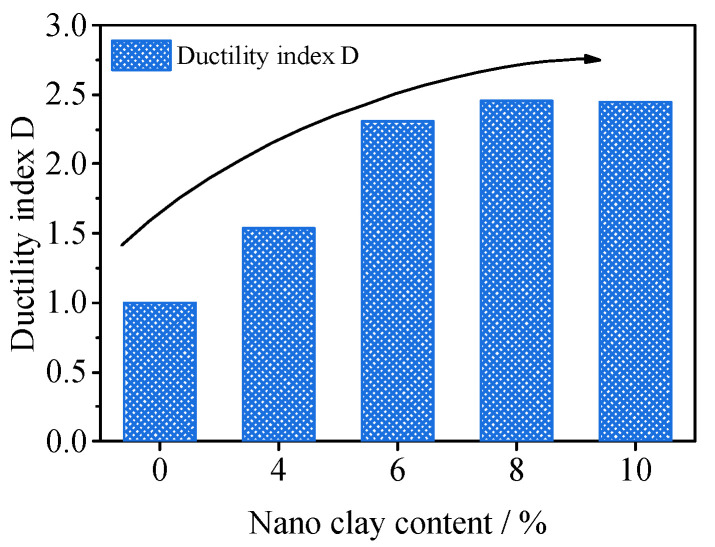
Relationship between nano-clay content and ductility index.

Ductility index D [28] is introduced to determine the ductility effect of nano-clay-reinforced nano-SiO_2_ and cement-solidified coastal soft soil (Formula 1). The link between nano-clay and ductility index D is depicted in Figure 4. It can be seen from the figure that the incorporation of nano-clay can improve the ductility index of SCSC samples. In the case of an increase in nano-clay content from 0% to 8%, the ductility index of the SCSC sample increases from 1 to 2.45. The ductility index of SCSC10 is similar to that of SCSC8, indicating that the ductility of the sample will not be improved when the nano-clay content reaches 8% in the range of 0–10%. The ductility index is often used in fiber-modified soil. Sung et al. [29] studied the use of PVA fiber to modify cement sand. The results showed that the ductility index of samples containing 1% PVA fiber was 3.5 times that of samples without PVA fiber. Bekhiti et al. [28] studied waste tire rubber fiber-modified cement bentonite. The results showed that the ductility index of 2% waste tire rubber fiber-modified cement bentonite was 5 times that of cement bentonite. The effect of nano-clay on soil ductility was weaker than that of fiber.

(1) $$\begin{array}{c} D=\frac{\Delta_{\mathrm{NC}}}{\Delta_{\mathrm{noNC}}} \end{array}$$

where ∆NC represents the axial strain corresponding to the unconfined compressive strength of the sample mixed with nano-clay, and ∆noNC represents the axial strain corresponding to the unconfined compressive strength of SCSC0.

### 3.2. Unconsolidated Undrained Shear Test

**Figure 5:**
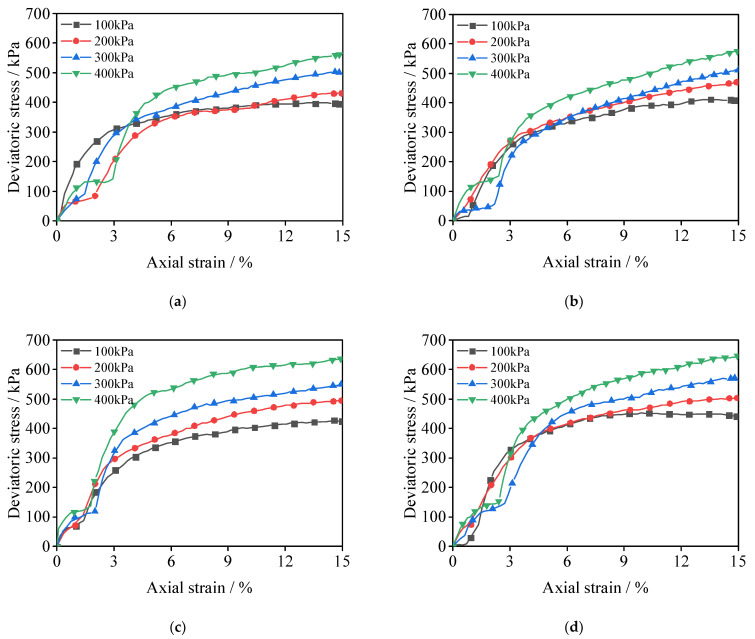

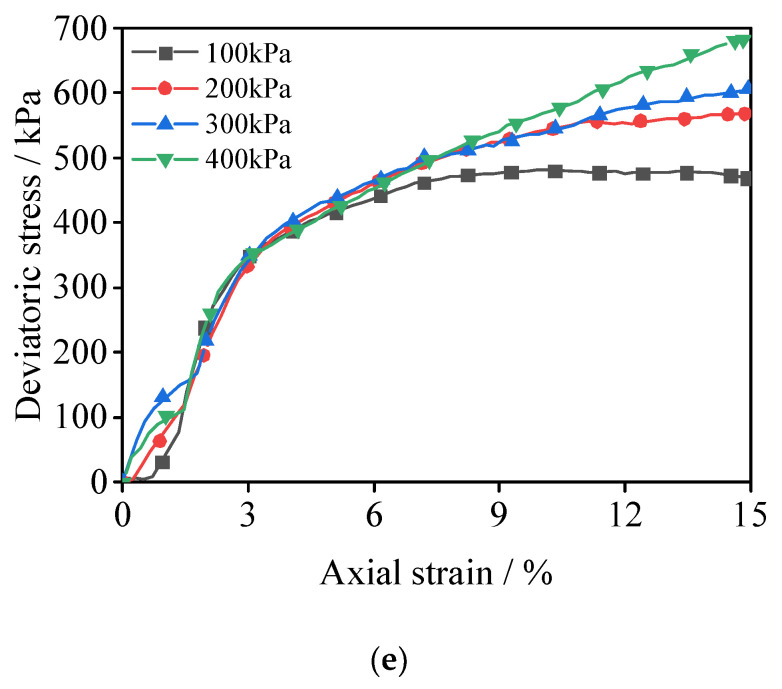
Deviatoric stress–strain curves of SCSC. (**a**) SCSC0 (**b**) SCSC4 (**c**) SCSC6 (**d**) SCSC8 (**e**) SCSC10.

A deviatoric stress–strain curve for SCSC samples is shown in Figure 5. Comparing the samples with different nano-clay contents in Figure 5a–e, with an increase in axial strain, the slope of the curve decreases, while the deviatoric stress increases continuously. The curve transitions from a softening trend to a hardening trend with increasing nano-clay doping, which indicates that the addition of nano-clay increases the toughness of the specimens significantly. With the increase in confining pressure, all stress–strain curves also show an increasing trend. Deviatoric stress increases with nano-clay content when confining pressure is increased. Taking the data under 100 kPa confining pressure as an example, the shear strength of SCSC4, SCSC6, SCSC8 and SCSC10 increased by 3.3%, 7.3%, 13.5% and 20.8% compared with that of SCSC0.

#### 3.2.1. Shear Index

**Figure 6:**
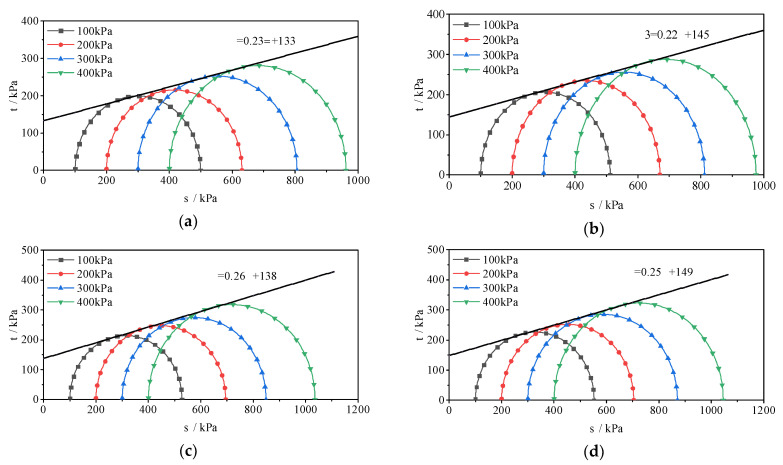

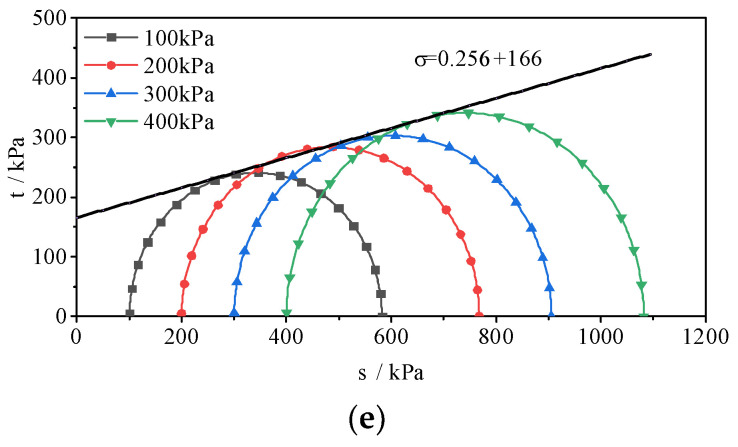
Mohr stress circle of SCSC. (**a**) SCSC0 (**b**) SCSC4 (**c**) SCSC6 (**d**) SCSC8 (**e**) SCSC10.

Based on the above analysis, nano-clay has a significant impact on the shear strength of nano-SiO_2_-reinforced cement soil. To further investigate how nano-clay affects SCSC shear resistance indices, the cohesion c and internal friction angle φ (Table 4) of SCSC samples are obtained by drawing the Mohr stress circle (Figure 6).

It can be seen from the table that the internal friction angle and cohesion of the sample show an overall upward trend after the incorporation of nano-clay. Compared with SCSC0, the internal friction angles of SCSC6, SCSC8 and SCSC10 increase by 12.5%, 8.4% and 8.4%, respectively, but that of SCSC4 decreases by 4.4%. This may be because the incorporation of nano-clay makes the clay particles effectively cemented, which limits the relative position of sand particles, and prevents effective sliding friction of soil during the shear process, thus reducing the friction in the soil [30]. Then, with the increase in nano-clay content, the friction angle in clay keeps increasing. Therefore, the increase in nano-clay content can significantly improve the shear strength parameters of the sample. The cohesion of SCSC samples with nano-clay is improved. The cohesion of SCSC4, SCSC6, SCSC8 and SCSC10 is 145 kPa, 138 kPa, 149 kPa and 166 kPa, respectively, which is 9.0%, 3.7%, 12.0% and 24.8% higher than that of SCSC0. The reason is that nano-clay can react with Ca(OH)_2_ produced by cement hydration to promote the production of CSH and CAH gels [31]. More gel materials can cement soil particles and improve the cohesion of soil.

#### 3.2.2. Duncan–Chang model

**Figure 7:**
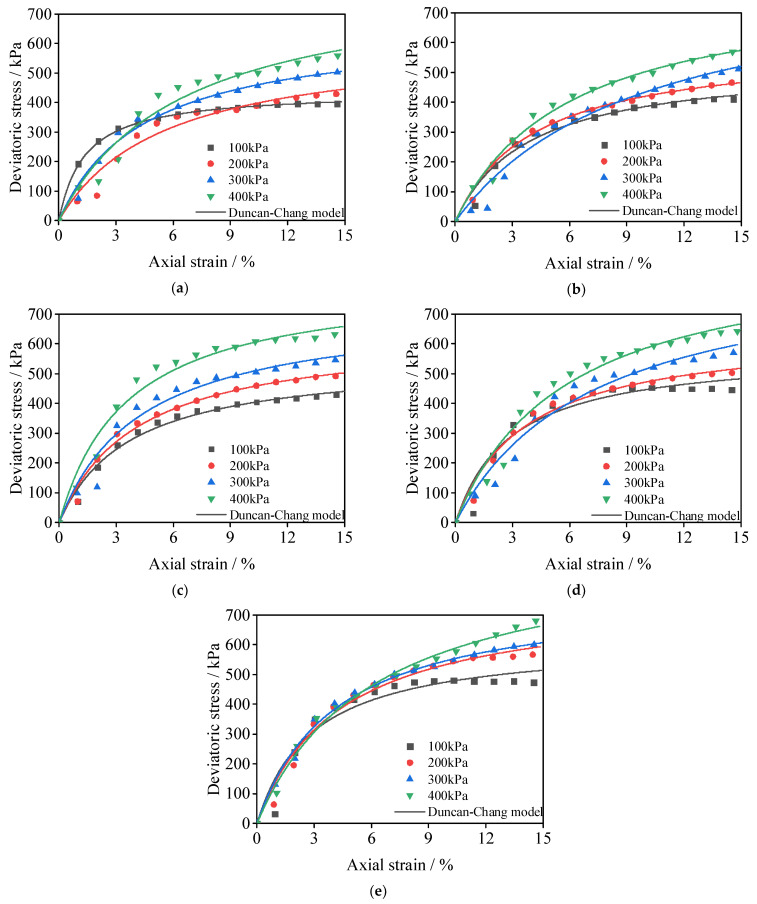
Comparison between Duncan–Chang model and measured results. (**a**) SCSC0 (**b**) SCSC4 (**c**) SCSC6 (**d**) SCSC8 (**e**) SCSC10.

Among soil constitutive models, the Duncan–Chang model is a typical nonlinear elastic model [32], that is primarily used for hardening the stress–strain curve in unconsolidated undrained triaxial tests. In this paper, the curve trend of the unconsolidated undrained shear test conforms to the hardening curve, so it is substituted into the Duncan–Chang model (Formula (2)). The Duncan–Chang model is highly fitted to the measured results (Figure 7), indicating that the nano-clay + nano-SiO_2_ + cement soil conforms to the nonlinear relationship shown in the model. The fitting parameters a and b are shown in Table 5.

(2) $$\begin{array}{c} \sigma_{1}-\sigma_{3}=\frac{\varepsilon_{1}}{a+b\varepsilon_{1}} \end{array}$$

Figure 7 shows the comparison between the Duncan–Chang model and measured results. As can be seen from the figure, the measured value has a slight deviation from the Duncan–Chang model when the axial strain is minor. This is because this test is an unconsolidated undrained test. At the beginning of shearing, the sample is in the pre-compression stage, and the contact between the sample and the axial load sensor is insufficient. When the shear continues, the sample is in complete contact with the axial load sensor, and the experimental results are highly consistent with the Duncan–Chang model, indicating that the characteristics of nano-clay + nano-SiO_2_ + cement soil at 7 days of curing conform to nonlinear characteristics. Table 5 shows the parameters of the Duncan–Chang model. It can be seen from the table that parameter a increases with the increase in confining pressure, and parameter b decreases with increasing constraint pressure. The incorporation of nano-clay makes the parameters a and b gradually decrease, indicating that the incorporation of nano-clay will harden the sample.

## 4. Conclusions

In this paper, the mechanical properties of cement-stabilized coastal soft soil modified by nano-clay and nano-SiO_2_ were studied through an unconfined compressive test and unconsolidated undrained shear test. The following conclusions are drawn:

(1) In the unconfined compression test, the addition of nano-clay will not change the softening curve characteristics of cement soil mixture. Nevertheless, when the addition of nano-clay can enhance the plasticity of cement-stabilized soil and increase the plastic failure strain, this mechanical behavior conforms to the requirements of engineering application.
(2) In the 0–10% nano-clay content, the unconfined compressive strength of the SCSC sample increases with the incorporation of nano-clay, and reaches the maximum unconfined compressive strength when the nano-clay content is 10%. Moreover, the ductility index of cement stabilized is also enhanced by nano-clay.
(3) In the unconsolidated undrained shear test, the deviatoric stress–strain curve of the samples exhibited the hardening curve. In the 0–10% nano-clay content, the shear strength increases with the increasing nano-clay content, reaching the maximum shear strength when the nano-clay content is 10%. Meanwhile, the deviatoric stress–strain curves of SCSC samples correlated with the Duncan–Chang model well.

## Figures and Tables

**Table 1:** Basic physical and mechanical properties of coastal soft soil.

Density (g/cm^3^)	Porosity (%)	Liquid Limit (%)	Plastic Limit (%)	Plastic Index
1.63	1.62	30.4	2.68	20.4

**Table 2:** Basic physical performance indexes of P.O32.5 cement.

Fineness (%)	Compressive Strength (MPa)		Flexural Strength (MPa)		Initial Setting Time (Min)	Final Setting Time (Min)
	3 d	28 d	3 d	28 d		
3.4	4.8	8.9	24.5	47.6	200	350

**Table 3:** Experiment plan.

Sample No.	Water Content (%)	Cement Content (%)	Nano-SiO_2_ Content (%)	Nano-Clay Content (%)	Curing Age (Days)
SCSC0	50	10	4.5	0	7
SCSC4				4	
SCSC6				6	
SCSC8				8	
SCSC10				10	

**Table 4:** Friction angle and cohesion of SCSC.

Shear Index	SCSC0	SCSC4	SCSC6	SCSC8	SCSC10
Friction angle φ (°)	12.95	12.41	14.57	14.04	14.04
Cohesion c (kPa)	133	145	138	149	166

**Table 5:** Fitting parameters of Duncan–Chang model.

Sample Number	Confining Pressure	A	B
SCSC0	100	0.0031	0.00228
	200	0.00908	0.00164
	300	0.00679	0.00152
	400	0.00775	0.0012
SCSC4	100	0.00721	0.00187
	200	0.00694	0.00168
	300	0.01136	0.00116
	400	0.00743	0.00125
SCSC6	100	0.00674	0.00182
	200	0.00637	0.00156
	300	0.00596	0.00138
	400	0.00438	0.00123
SCSC8	100	0.00506	0.0073
	200	0.00563	0.00155
	300	0.00813	0.00113
	400	0.00627	0.00108
SCSC10	100	0.00475	0.00163
	200	0.00536	0.00141
	300	0.00587	0.00129
	400	0.00659	0.00107

## Data Availability

Not applicable.

## References

[1] Wang J., Zhou Z., Fu H., Dong Q., Cai Y., Hu X. (2019). Influence of vacuum preloading on vertical bearing capacities of piles installed on coastal soft soil. Mar. Geores. Geotechnol..

[2] Dongxing W., Zentar R., Abriak N.E. (2016). Interpretation of compression behavior of structured and remolded marine soils. J. Mater. Civ. Eng..

[3] Mohamad N.O., Razali C.E., Hadi A.A.A., Som P.P., Eng B.C., Rusli M.B., Mohamad F.R. (2016). Challenges in Construction Over Soft Soil—Case Studies in Malaysia. IOP Conf. Ser. Mater. Sci. Eng..

[4] Idrus M.M.M., Singh J.S.M., Musbah A.L.A., Wijeyesekera D.C. (2016). Investigation of Stabilised Batu Pahat Soft Soil Pertaining on its CBR and Permeability Properties for Road Construction. IOP Conf. Ser. Mater. Sci. Eng..

[5] Eisa M.S., Basiouny M.E., Mohamady A., Mira M. (2022). Improving Weak Subgrade Soil Using Different Additives. Materials.

[6] Basha E.A., Hashim R., Mahmud H.B., Muntohar A.S. (2005). Stabilization of residual soil with rice husk ash and cement. Constr. Build. Mater..

[7] Ali F.H., Adnan A., Choy C.K. (1992). Geotechnical properties of a chemically stabilized soil from Malaysia with rice husk ash as an additive. Geotech. Geol. Eng..

[8] Fang J., Wang Y., Wang K., Dai W., Yu Y., Li C. (2022). Experimental Study on the Mechanical Properties of Diatomite-Modified Coastal Cement Soil. Materials.

[9] Yousefi Oderji S., Chen B., Ahmad M.R., Shah S.F.A. (2019). Fresh and hardened properties of one-part fly ash-based geopolymer binders cured at room temperature: Effect of slag and alkali activators. J. Clean. Prod..

[10] Yao K., Wang W., Li N., Zhang C., Wang L. (2019). Investigation on strength and microstructure characteristics of nano-MgO admixed with cemented soft soil. Constr. Build. Mater..

[11] Puppala Anand J., Hoyos Laureano R., Potturi Ajay K. (2011). Resilient Moduli Response of Moderately Cement-Treated Reclaimed Asphalt Pavement Aggregates. J. Mater. Civ. Eng..

[12] Zhang T., Cai G., Liu S. (2018). Reclaimed Lignin-Stabilized Silty Soil: Undrained Shear Strength, Atterberg Limits, and Microstructure Characteristics. J. Mater. Civ. Eng..

[13] Bhanja S., Sengupta B. (2005). Influence of silica fume on the tensile strength of concrete. Cem. Concr. Res..

[14] Xiaokang Z., Qiao D., Xueqin C., Xingyu G., Liyuan W. (2020). Mesoscale Cracking of Cement-treated Composites with Initial Defects. China J. Highw. Transp..

[15] Yu W., Li N., Dai M., An D., Qian B., Wang W., Jiang P. (2021). Consolidation Behavior and Compression Prediction Model of Coastal Cement Soil Modified by Nanoclay. Adv. Mater. Sci. Eng..

[16] Wei W., Jingjing L., Na L., Lu M. (2022). Mechanical properties and micro mechanism of nano-SiO_2_ modified coastal cement soil at short age. Acta Mater. Compos. Sin..

[17] Majeed Z.H., Taha M.R. (2012). Effect of Nanomaterial Treatment on Geotechnical Properties of a Penang Soft Soil. J. Asian Sci. Res..

[18] Li Z., Zhao Z., Shi H., Li J., Zhao C., Wang P. (2022). Experimental Study on PVA-MgO Composite Improvement of Sandy Soil. Materials.

[19] Qing Y., Zenan Z., Li S., Rongshen C. (2006). A comparative study on the pozzolanic activity between nano-SiO_2_ and silica fume. J. Wuhan Univ. Technol.-Mat. Sci. Edit..

[20] Zhuang C., Chen Y. (2019). The effect of nano-SiO_2_ on concrete properties: A review. Nanotechnol. Rev..

[21] Zhenjiang X., Menghui L., Rong C., Fuhai L., Xuan Y., Qingxu Z. (2022). Research on the inhibition of efflorescence of slab ballastless track by using nano silica. Railw. Stand. Des..

[22] Jie L., Xudong S., Wenfu H. (2022). Research on the macro and micro performance of concrete under the action of different nano SiO_2_ particle size. Concr. Eng. Int..

[23] Mirgozar Langaroudi M.A., Mohammadi Y. (2018). Effect of nano-clay on workability, mechanical, and durability properties of self-consolidating concrete containing mineral admixtures. Constr. Build. Mater..

[24] Hamed N., El-Feky M.S., Kohail M., Nasr E.-S.A.R. (2019). Effect of nano-clay de-agglomeration on mechanical properties of concrete. Constr. Build. Mater..

[25] Zhao Y., Ling X., Gong W., Li P., Li G., Wang L. (2020). Mechanical Properties of Fiber-Reinforced Soil under Triaxial Compression and Parameter Determination Based on the Duncan-Chang Model. Appl. Sci..

[26] Gao M., Jin X., Zhao T., Li H., Zhou L. (2022). Study on the strength mechanism of red clay improved by waste tire rubber powder. Case Stud. Constr. Mater..

[27] Wang W., Li J., Hu J. (2020). Unconfined Mechanical Properties of Nanoclay Cement Compound Modified Calcareous Sand of the South China Sea. Adv. Civ. Eng..

[28] Bekhiti M., Trouzine H., Rabehi M. (2019). Influence of waste tire rubber fibers on swelling behavior, unconfined compressive strength and ductility of cement stabilized bentonite clay soil. Constr. Build. Mater..

[29] Park S.-S. (2011). Unconfined compressive strength and ductility of fiber-reinforced cemented sand. Constr. Build. Mater..

[30] Longwei W., Jin L., Lingzhi X., Lilin W., Chuan Z., Changqing Q. (2020). Triaxial shear test of sand improved by polymer composite. Hydrogeol. Eng. Geol..

[31] Niu X.-J., Li Q.-B., Hu Y., Tan Y.-S., Liu C.-F. (2021). Properties of cement-based materials incorporating nano-clay and calcined nano-clay: A review. Constr. Build. Mater..

[32] Duncan James M., Chang C.-Y. (1970). Nonlinear Analysis of Stress and Strain in Soils. J. Soil Mech. Found. Div..

